# Electron Paramagnetic Resonance and Electron Spin Echo Studies of Co^2+^ Coordination by Nicotinamide Adenine Dinucleotide (NAD^+^) in Water Solution

**DOI:** 10.1007/s00723-013-0444-z

**Published:** 2013-02-24

**Authors:** Stanisław K. Hoffmann, Janina Goslar, Stefan Lijewski

**Affiliations:** Institute of Molecular Physics, Polish Academy of Sciences, Smoluchowskiego 17, 60-179 Poznan, Poland

## Abstract

Co^2+^ binding to the nicotinamide adenine dinucleotide (NAD^+^) molecule in water solution was studied by electron paramagnetic resonance (EPR) and electron spin echo at low temperatures. Cobalt is coordinated by NAD^+^ when the metal is in excess only, but even in such conditions, the Co/NAD^+^ complexes coexist with Co(H_2_O)_6_ complexes. EPR spin-Hamiltonian parameters of the Co/NAD^+^ complex at 6 K are *g*_*z*_ = 2.01, *g*_*x*_ = 2.38, *g*_*y*_ = 3.06, *A*_*z*_ = 94 × 10^−4^ cm^−1^, *A*_*x*_ = 33 × 10^−4^ cm^−1^ and *A*_*y*_ = 71 × 10^−4^ cm^−1^. They indicate the low-spin Co^2+^ configuration with *S* = 1/2. Electron spin echo envelope modulation spectroscopy with Fourier transform of the modulated spin echo decay shows a strong coordination by nitrogen atoms and excludes the coordination by phosphate and/or amide groups. Thus, Co^2+^ ion is coordinated in pseudo-tetrahedral geometry by four nitrogen atoms of adenine rings of two NAD^+^ molecules.

## Introduction

**Fig. 1:**
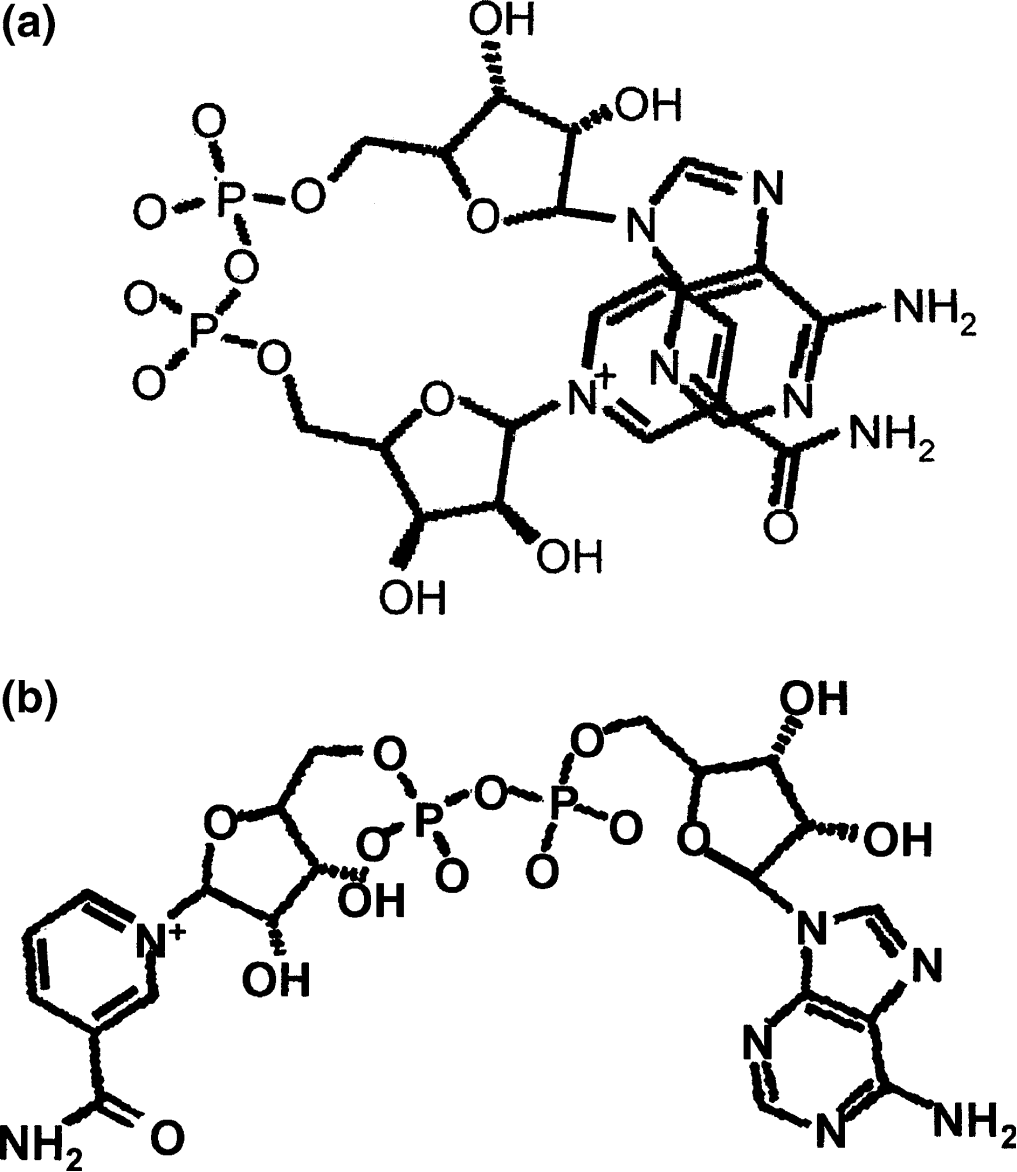
Molecular structure of NAD^+^ in: **a** the folded (compact) and **b** extended (open) conformation

Nicotinamide adenine dinucleotide (NAD^+^) molecule consists of two nucleotides joined by two bridging phosphate groups. The nucleotides consist of ribose rings with one nucleotide containing adenosine base and the other containing nicotinamide (Fig. 1). NAD^+^ plays several essential roles in metabolism of living organisms where it appears as *β*-diastereomer. It acts as a coenzyme in redox reactions of cellular respiration and is involved in an intermolecular electron transfer. NAD^+^ is a donor of ADP ribosylation reaction and acts as a substrate for bacterial DNA ligases [1]. NAD^+^ is very flexible molecule with over a dozen rotatable bonds that can adopt a wide variety of environmentally dependent conformations [2, 3]. The two extreme NAD^+^ conformations are a folded and an extended configuration. The folded (compact) conformer (Fig. 1a), having aromatic rings in close proximity, exists in water solutions where it reduces solvation accessible molecular surface area. The folded conformation also appears in a single crystal of nicotinamide adenine dinucleotide tetrahydrate both at room [4] and at low temperature (100 K) [5] and is stabilized by hydrogen bonds to the crystalline water molecules. NAD^+^ bound to enzyme adopts an extended (open) conformation (Fig. 1b) allowing a catalytic activity by forming weak hydrogen bonds between an enzyme and the cofactor [6, 7]. Extended configuration is preferred for a free NAD^+^ molecule as indicated by DFT calculations [8].

NAD^+^ molecule is a potential binder of metal ions. Coordinated metal ions can modify the electron density distribution over the molecule; it can also influence a possibility of configuration changes, as well as the dynamics of coenzyme binding what may be essential for its biochemical activity in the Wilson disease or a poisoning. There exist four potential sites of metallation in NAD^+^ molecule: oxygen atoms of the phosphate groups; the amide group of the nicotinamide moiety, oxygens of the ribose molecules and nitrogens of adenosine. On the basis of kinetic, potentiometric and calorimetric studies, it was suggested that various metal ions prefer coordination at different sites.

Ni^2+^ ions are coordinated by phosphate moiety and interact simultaneously with adenine and nicotinamide rings of NAD^+^ as inferred from temperature–jump relaxation studies [9–11]. The VO^4+^ ion acts as a diphosphate chelator in the acidic range of water solution, whereas in the basic range, a binding of the ion to the deprotonated hydroxyls of two ribose moieties was suggested [12]. The Mn^2+^ ion was found as chelated by N and O of the nicotinamide group [13] and as bonded to oxygens of all the phosphates in phosphate NAD (NADP) [14]. Cr^5+^ ions were identified as oxygen bonded to the NAD^+^ [15]. A coordination of Mg^2+^ to a hydroxyl group of the ribose was studied by the density functional theory (DFT) calculations [8]. Cobalt ion complexation by NAD^+^ was studied by calorimetry and potentiometry methods, and it was suggested that similarly to Ni^2+^, the Co^2+^ ions can be chelated by oxygens of the phosphorous group and nitrogens of the adenosine ring [11]. The above-mentioned coordination modes need a confirmation by a microscopic method as, for example, the electron paramagnetic resonance (EPR) or electron spin echo envelope modulation (ESEEM) spectroscopy, which are able to identify the ligand atoms around a paramagnetic central ion. Such investigations we have recently performed for identification of Cu^2+^ binding sites in NAD^+^ in water solutions [16]. We have shown that Cu^2+^ is coordinated by two hydroxyl oxygen atoms of ribose moieties of two NAD^+^ molecules and four solvated H_2_O molecules forming axially deformed octahedral chromophore [CuO_2_(H_2_O)_4_].

In this paper, we describe the coordination site and a model of Co/NAD^+^ complex resulting from EPR and electron spin echo (ESE) methods.

## Methods

### Materials

Nicotinamide adenine dinucleotide (*β*-NAD^+^) used in the experiments was purchased from Sigma without additional purification. Cobalt nitrate (from POCh Gliwice, Poland) was used after double crystallization from water. All the solutions were prepared with deionized and then distilled water for various pH values with excess of ligand or excess of metal with metal ions concentration in the range 8.5–9.0 × 10^−3^ M. We have found that above pH 8.2 a poorly soluble pink color precipitation occurs and the best NAD^+^ coordination conditions exist in the samples with excess of metal. Thus, we present the EPR results for samples with the metal-to-ligand concentration ratio M:L = 4.5:1, with *C*_Co_ = 9 × 10^−3^ M, *C*_NAD_ = 2 × 10^−3^ M.

### EPR spectroscopy

EPR and ESE measurements were performed for the Co/NAD^+^ system using a Bruker ESP380E FT/CW spectrometer with a loop-gap resonator equipped with a helium flow Oxford CF935 cryostat. EPR and ESE measurements were done at low temperatures (4–10 K) for frozen solutions using glycerol/water solvent. This solvent allowed obtaining uniform glasses after rapid freezing at liquid nitrogen. Even at such low temperature, a saturation effect was easily visible, thus to avoid this effect, we recorded the spectra at high microwave power attenuation of 45 dB (0.007 mW). The EPR spectrum was simulated using the Bruker SimFonia routine. In pulsed EPR experiments, the ESE signal was used for recording the echo-detected (ED) EPR spectra and for observations of the two- and three-pulse ESE decay. The ED EPR spectra are obtained when the ESE amplitude is recorded during magnetic field sweep through the EPR spectrum. The two-pulse ESE signal was excited by two 24 ns pulses (excitation bandwidth of 1.8 mT) with interpulse interval *τ* = 96–176 ns at 272 mT. The maximal echo amplitude was observed for *τ* = 96 as expected for echo decay modulated by protons. The stimulated ESE was generated by three 24 ns pulses with first interpulse interval *τ* = 96–176 ns and varied the second interval *T* starting from 96 ns with 8 ns step.

## Results and discussion

### EPR of Co^2+^ ions

**Fig. 2:**
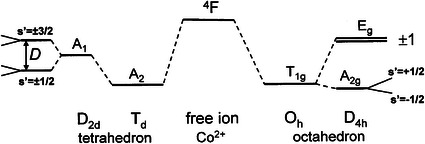
Ground state level splitting of Co^2+^ (*S* = 3/2) complexes in octahedron *O*_h_ and tetrahedron *T*_d_ with the axial deformation to *D*_4h_ and *D*_2d_ symmetry, respectively, with the final Zeeman splitting

An analysis of Co^2+^ EPR spectra is not straightforward because the spectra can be recorded usually at low temperatures because of a very fast electron spin–lattice relaxation which produces strong EPR line broadening. Moreover, Co^2+^ ions (3d^7^ configuration with *S* = 3/2, *I* = 7/2) can appear both at high-spin (*S* = 3/2) and at low-spin (*S* = 1/2) configurations and have different characteristics in tetrahedral and octahedral coordination. The ground state term ^4^F of Co^2+^ is split by the electric ligand field giving the orbital triplet ground state *T*_1g_ in octahedral *O*_h_ symmetry and the orbital singlet ground state *A*_2_ in tetrahedral *T*_d_ symmetry. Tetragonal distortion *D*_4h_ of the regular symmetry leads to the triplet splitting into a singlet and a doublet in an octahedron, whereas the *A*_1_ singlet in the tetrahedral environment undergoes a zero-field splitting *D* usually with effective spin *S*′ = ½ (when *D* > hν) as it is shown in Fig. 2. Finally, the ground orbital states in axially distorted octahedron or tetrahedron are Kramers doublets split in the external magnetic field. Both the octahedral and the tetrahedral Co^2+^ complexes exist, but the tetrahedral coordination seems to be preferred.

EPR spectra and spin-Hamiltonian parameters are well understood for high-spin Co^2+^ in ideal high crystal field symmetries and at the tetragonal distortion [17, 18]. The theory for octahedral field *O*_h_ predicts that the EPR spectrum is isotropic with *g* = 4.33 [19]. This value markedly differs from true *g*-factors which are close to *g* = 2. This is due to considerable excited orbital contributions and spin–orbit coupling. As a consequence, the *g*-values are extremely sensitive to the distortion of the octahedral environment and vary in the range *g* = 2–9. The characteristic feature of the deformed octahedral field is the sum of the three *g*-factors being *g*_||_ +2 *g*_⊥_ = 3 × 4.33 = 13 or *g*_*x*_ + *g*_*y*_ + *g*_*z*_ ≈ 13. The sequence *g*_||_ > *g*_⊥_ exists when the E_g_ state is the lowest (compressed octahedron), whereas *g*_||_ > *g*_⊥_ when the *A*_2g_ state has the lowest energy (elongated CoX_6_).

For high-spin Co^2+^ in the distorted tetrahedral geometry, either the ±1/2 state or the ±3/2 state can be lower in energy [20, 21]. The ±1/2 state is lower in a flattened tetrahedron, and the ±3/2 state is lower in an elongated tetrahedron of *D*_2d_ symmetry. The true *g*-factors are expected in the range 2.2–2.4, whereas the *g*-factors for the effective spin *S*′ = 1/2 vary from 2 to 6, and their sum is usually *g*_*x*_ + *g*_*y*_ + *g*_*z*_ ≈ 8 for a small axial distortion. The hyperfine splitting, when it appears in the EPR spectrum, is usually smaller in tetrahedral complexes (*A*_*z*_ ≈ 100 × 10^−4^ cm^−1^) than that in octahedral complexes (*A*_*z*_ ≈ 200 × 10^−4^ cm^−1^).

The low-spin Co^2+^ complexes with *S* = 1/2 often appear for the square planar and in pseudo-tetrahedral geometry [22], and they are easily distinguished from the high-spin complexes. The *g*-factors are in the range 1.5–3.3 with *g*_*x*_ + *g*_*y*_ + *g*_*z*_ ≈ 6–7, and ground state strongly depends on the crystal field strength, geometry and mixing of configurations [17, 23]. It is clear from the above that it is possible to distinguish between octahedral and tetrahedral complex geometry and the spin state of Co^2+^ considering the sum of the *g*-factors only.

### EPR spectra of Co/NAD^+^

**Fig. 3:**
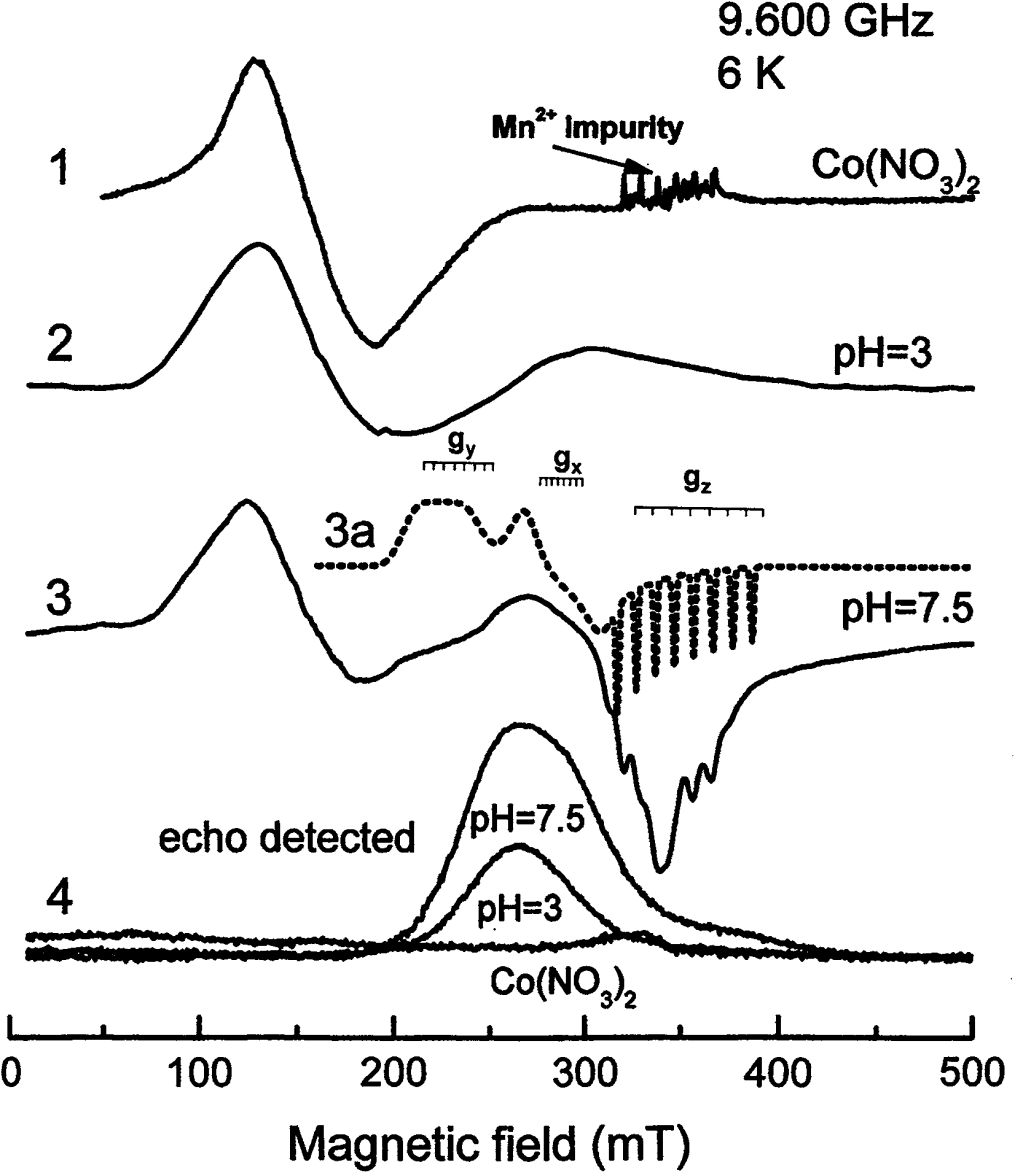
Low-temperature EPR spectra recorded at low microwave power: *1* Co(NO_3_)_2_ recorded as a reference signal; *2* Co/NAD^+^ frozen solution at pH = 3; *3* Co/NAD^+^ frozen solution at pH = 7.5. The *dotted line* (3a) is the simulated spectrum with *stick diagrams* of eight-component hyperfine structure of Co^2+^ (*I* = 7/2); *4* corresponding echo-detected spectra. The line marked as *g*_iso_ arises from Co(H_2_O)_6_ complexes

EPR spectra of Co/NAD^+^ system were recorded at 6 K. At lower temperatures, the saturation effect strongly deforms EPR lines even for low incident microwave power, whereas at higher temperatures, the lines become broadened, especially the single broad line at about 150 mT (*g* = 4.3). The spectra were recorded using the continuous-wave technique (cw-EPR) which gives the first derivative of the absorption as well as by the ED ESE technique which gives an absorption line. These spectra are compared in Fig. 3. Additionally, we have recorded EPR spectrum of Co(NO_3_)_2_ in glycerol-water solution (spectrum 1 in Fig. 3) for a comparison with Co/NAD^+^ spectra. The Co(NO_3_)_2_ spectrum contains a strong broad signal at *g* = 4.3 (about 150 mT). This spectrum is typical for high-spin Co^2+^ ions (*S* = 3/2) in the octahedral coordination and characteristic for Co(H_2_O)_6_ complexes [24]. Six narrow weak lines in this spectrum around *g* = 2 (330 mT) are due to Mn^2+^ impurities.

At low pH values (below 4.5), the EPR spectrum (spectrum 2 in Fig. 3) is dominated by the low-field line as in the spectrum 1. It indicates that at low pH values, mostly the hexa-aqua Co^2+^ complexes are observed. A weak broad absorption line visible around 300 mT of spectrum 2 can be due to Co^2+^ ions coordinated by NAD^+^ molecules. This line grows in amplitude for higher pH values where it is clearly visible as anisotropic EPR spectrum with resolved peaks at principal *g*-values with weakly resolved hyperfine splitting. This anisotropic spectrum coexists with the line at *g* = 4.3 and it is shown as spectrum 3 in Fig. 3. The EPR *g*-factors of the spectrum and corresponding hyperfine splitting *A* were determined by computer simulations. The simulated spectrum is shown by the dotted line accompanying spectrum 3 with stick diagrams representing eight hyperfine (*I* = 7/2 for ^59^Co of 100 % abundance) lines around the *g*-factor fields. The individual hyperfine lines have peak-to-peak line width of about 2 mT. In the simulated spectrum, much lower line width was assumed around *g*_*z*_ to show clearly the octet hyperfine line positions in the experimental spectrum. It is visible that the anisotropic spectrum with the resolved structure is superimposed on the broad structureless line resulting from complexes having fast spin relaxation or having distributed spin-Hamiltonian parameters. The spin Hamiltonian of the Co/NAD^+^ complex *g*-factors and hyperfine splittings obtained from computer simulations are as follows: *g*_*z*_ = 2.01, *g*_*x*_ = 2.38, *g*_*y*_ = 3.06, *A*_*z*_ = 94 × 10^−4^ cm^−1^ (10 mT), *A*_*x*_ = 33 × 10^−4^ cm^−1^ (3 mT) and *A*_*y*_ = 71 × 10^−4^ cm^−1^ (5 mT). The parameters clearly indicate the low-spin Co^2+^ configuration with *S* = 1/2 which is expected in the strong crystal field mainly of the pseudo-tetrahedral, close to the square-planar geometry [17, 22].

### Echo-detected spectra of Co/NAD^+^

Echo-detected spectra deliver new information. In Co(NO_3_)_2_ frozen water solution, an ESE signal cannot be generated. We check this for different interpulse interval in the range *τ* = 96–176 ns to avoid blind spots due to echo amplitude modulations. No trace of the echo signal we have also found in the ED spectrum of Co(H_2_O)_6_ (spectra 4 in Fig. 3). It indicates that the *g* = 4.3 line is homogeneously broadened and does not participate in the ESE formation. The homogeneous line broadening can be due to the fast spin-lattice relaxation. In ED spectra of Co/NAD^+^ systems, the line around *g* = 4.3 also does not exist and the broad absorption band around 300 mT confirms that the weak absorption in spectrum 2 is due to the same complexes as for the higher pH value (spectrum 3).

It seems that not all Co^2+^ species give signal in EPR spectrum. Intensity of the Co(H_2_O)_6_ line and low-spin Co/NAD^+^ line are nearly the same, suggesting that the some number of Co^2+^ ions are involved in these two forms of coordination. However, there exist excess of metal ions over the NAD^+^ molecules (4.5:1), and one can expect that when all NAD^+^ molecules will coordinate Co^2+^ ions then the intensity of the Co(H_2_O)_6_ line should be four times larger when mono-NAD^+^ adduct is formed or eight time larger when two NAD^+^ coordinate single Co^2+^ ion. Thus, other Co/NAD^+^ species seems to be formed in the solution, as Co(NAD^+^)(H_2_O)_5_, Co(NAD^+^)_2_(H_2_O)_4_ or Co(NAD^+^)_2_(H_2_O)_2_, but they are EPR silent or give very broad and weak absorption lines.

### Electron spin echo envelope modulations

**Fig. 4:**
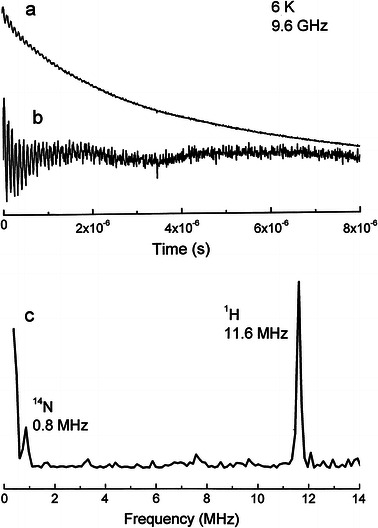
Electron spin echo decay (**a**), its modulation (**b**), and Fourier transform of modulation function (**c**) showing peaks from coordinated nitrogen atoms and distant hydrogen atoms. The high-intensity line near zero frequency in the trace (**c**) is an artifact due to a non-perfect baseline correction in the modulation function

Conclusive information on the Co^2+^ coordination site can be obtained from Fourier transform pulse EPR experiments. We recorded the two-pulse Hahn echo and three-pulse stimulated ESE amplitude decay for the echo signal generated by microwave pulses at magnetic field 272.1 mT close to the *g*_*x*_-value field. The three-pulse ESE signal decays with time as it is shown in Fig. 4a. The ESE amplitude decays relatively fast with the characteristic time *T*_d_ ≈ 5 μs, close to the spin–lattice relaxation time. The decay is modulated by the magnetic dipolar interaction with surrounding magnetic nuclei. The modulation pattern subtracted from the decay is shown as trace “b” in Fig. 4. The Fourier transform of the modulation function displays peaks at frequencies of nuclear transition in the ESEEM, electron-nuclear double resonance-type spectrum (Fig. 4, trace “c”). The amplitude of the peaks in three-pulse ESEEM spectra of multinuclear spin systems (in our case ^1^H, ^14^N and ^31^P) can be affected by a peak suppression effect [25]. The basic peaks can be reduced in intensity even down to almost complete cancellation since their amplitude depends not only on their modulation depth but also on the modulation depth and blind spots of the other nuclei. The amplitude of peaks not necessarily reflects the relative number of interacting nuclei and can be controlled by varying the interpulse interval *τ.* Thus, we have taken ESEEM spectra for various interpulse intervals. The spectrum shown as trace “c” in Fig. 4 indicates a structureless peak at 11.6 MHz due to the dipolar coupling to distant matrix protons and a structureless peak at 0.8 MHz from the coupling to ^14^N nuclei as expected at magnetic field *B* = 272.1 mT. The fact that the ^14^N peak is observed at the Larmor frequency without line splitting indicates that the quadrupole interaction is relatively weak due to a small local crystal field gradient at nitrogen site. Such situation can be expected for the N atom in the adenosine rings, whereas for nitrogen nuclei in the nicotinamide NH_2_ group, large field gradient can be expected.

There is no peak from ^31^P nuclei of the phosphate groups, neither for two-pulse decay nor for three-pulse decay measured for various *τ*, which is expected at about 4.7 MHz. The phosphorous peaks have been identified in various materials where the ^31^P was located at the distance shorter than of about 0.5 nm [26–29]. Thus, ESEEM spectroscopy excludes the coordination of Co^2+^ to oxygen atoms of PO_4_ groups and strongly indicates the coordination by nitrogen atoms.

**Fig. 5:**
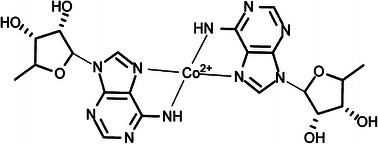
Proposed structure of Co^2+^ coordination by nitrogen of adenosine rings of two NAD^+^ molecules

The above results allow us to propose a model of coordination as it is shown in Fig. 5. The pseudo-tetrahedral or nearly planar coordination of Co^2+^ to nitrogen adenosine rings of two NAD^+^ molecules gives rise to the strong crystal electric field at the metal site allowing pairing of two spins of cobalt ions, thus giving the low-spin Co^2+^ ion. Our results confirm a suggestion drawn form potentiometric measurements that the Co^2+^ ion is preferentially coordinated to the adenine ring [11]. The similar low-spin complexes with a coordination by four nitrogen atoms were found in pyrazole, macrocyclic and porphyrine Co^2+^ complexes [30–32], and such a type of coordination was suggested in studies of adenine Co^2+^ complexes [17, 33, 34].

## Conclusions

The NAD^+^ molecule having a few potential binding sites is not very effective in binding of Co^2+^ ions in water solution. With excess of ligand and when pH of solution is lower than five, cobalt ions are coordinated by water molecules as indicated by EPR methods. Conditions for Co^2+^ coordination appear at higher pH values (easier deprotonation of ligand compensating excess positive charge of metal ion) with excess of metal in the solution. But even in such conditions, only a part of Co^2+^ ions is coordinated by NAD^+^, forming the low-spin pseudo-tetrahedral coordination to nitrogens of the adenosine rings of two NAD^+^ molecules, whereas the other Co^2+^ ions form the hexa-aqua or EPR-silent Co/NAD^+^ complexes. The Co^2+^ behavior is different from that we have found for Cu^2+^ at the same experimental conditions [16]. Practically, all Cu^2+^ ions were coordinated by NAD^+^ molecules in the whole pH range with chromophore [CuO_2_(H_2_O)_4_] where only two hydroxyl oxygens of two ribose moieties are involved. Thus, Co^2+^ and Cu^2+^ are coordinated in different sites of NAD^+^ molecules. It suggests a possibility of selective blocking of two different parts of the NAD^+^ molecules by paramagnetic ions.
